# Barriers to Rural Midwifery: An Integrative Review

**DOI:** 10.1111/jan.16737

**Published:** 2025-01-02

**Authors:** Madison Friesen, Viviane Josewski, Caroline Sanders

**Affiliations:** ^1^ School of Nursing University of Northern British Columbia, Northern Health Prince George British Columbia Canada; ^2^ School of Nursing University of Northern British Columbia Prince George British Columbia Canada

**Keywords:** midwifery, midwifery integration, midwifery sustainability, rural health, rural health access

## Abstract

**Aim:**

To explore the types of barriers that midwives face when practicing or attempting to practice in rural and remote locations.

**Design:**

An integrative review using the Ecological Systems Theory.

**Methods:**

The review was guided by Whitmore and Knafl. Included studies were appraised using the Mixed Methods Appraisal tool.

**Data Sources:**

In January 2024, searches were undertaken in CINHAL, MEDLINE, Science Direct, and Google Scholar.

**Results:**

A total of 470 articles were screened after searches. Fourteen articles published between 1990 and 2023 met all inclusion criteria. They were thematically analysed to explore barriers present at the micro‐, macro‐, and meso‐levels. The mico‐level barriers included isolation, financial insecurity due to low volume, and challenges in separating personal and professional life. Barriers at the meso level included discord in interprofessional relationships and challenges in attending continuing education. Lack of midwifery representation, overt medical dominance, and policy acted as barriers at the macro level.

**Conclusion:**

Rural midwives face complex challenges that demand multi‐faceted and multi‐level solutions. The findings highlight the need for an increase in midwifery representation in healthcare planning, improved policies related to midwifery, and the adoption of a rural model of healthcare planning that accounts for the unique social realities of living and practicing in rural communities.

**Implications for the Profession and Patient Care:**

By illuminating the challenges faced by rural midwives, efforts can be directed toward sustainable solutions to support rural midwifery practices and decrease rural health disparities.

**Impact:**

Increasing midwifery access in rural communities can help reduce maternity care disparities for rural families. By identifying and addressing the barriers experienced by rural midwives, it can strengthen advocacy for targeted policies and support systems that empower midwives.

**Reporting Method:**

This review is reported according to the PRISMA guidelines for scoping reviews.

**Patient or Public contribution:**

No Patient or Public Contribution.

**Summary**

- What is known?
  ○ Midwifery has the potential to support the sustainability of maternity care services in rural and remote communities.
  ○ In countries with well‐established midwifery, recruiting and retaining rural midwives is a known challenge.
  ○ Recruiting and retaining midwives in rural communities is especially challenging in countries where midwifery is less integrated into the healthcare system.
- What this paper adds?
  ○ Rural midwives encounter distinct challenges in their immediate environment, personal relationships, and interactions with more dominant providers, affecting their ability to integrate and sustain rural practice.
  ○ Biomedical dominance affects other healthcare professionals' understanding and acceptance of midwifery.
  ○ Leadership roles in rural midwifery practice have the potential to strengthen connections and collaborations that can support relational practice with other professional groups and advance policy development.
- Implications for practice/policy
  ○ Midwives must be supported to work to their full scope of practice alongside other healthcare professionals.
  ○ Enhancing midwifery representation in leadership and health planning committees can ensure that midwifery services are effectively integrated into rural healthcare systems.
  ○ When considering rural needs, rural planning committees must move away from overt biomedical urban healthcare models to support the integration of rural midwifery.

## 1 Introduction

Midwifery is often regarded as the first holistic profession, with a history that stretches back to the Palaeolithic era (Barnawi, Richter, and Habib 2013; International Confederation of Midwives 2022). With the medicalisation of childbirth, birth became dominated by physicians, and in the 19th century midwives had to struggle to retain their profession and advance their credentials through formal education and certification (Brodsky 2008). Nevertheless, midwifery has endured and continues to be a prominent profession in many countries, such as the United Kingdom, Netherlands, and New Zealand (Dawson et al. 2022; Gilkison et al. 2018; Weigers and Hukkelhoven 2010). In countries like the United States, Canada, and Australia, where childbirth is primarily governed by a medical model, midwives often encounter structural and functional limitations in their status and role (Fahy 2007; Kristienne McFarland et al. 2020). For example, in the United States, only a few jurisdictions fully integrate midwives as regulated health professionals into care provider networks (Vedam et al. 2018). A similar situation is observed in Australia, where, as Watkins et al. (2023) highlight, fragmented care services, medical dominance, and low status of midwifery within organisations prevent most midwives from working to their full scope of practice. Despite these challenges, a growing interest in midwifery has paved the way for the re‐emergence of the profession (Center For Women's Health 2024). Midwifery offers the potential to support the sustainability of maternity care in underserved areas, such as rural communities (Stoll and Kornelsen 2014). Yet, in countries with well‐established midwifery such as New Zealand and the United Kingdom, accessible, equitable maternity care for rural birthing persons remains a challenge (Dawson et al. 2022; Hoang, Quynh, and Ogden 2014).

Midwifery care was formally reintroduced within Canada in Ontario in 1994 (CAM 2023). Since then, midwifery has been implemented and regulated across Canada, with Prince Edward Island (PEI) being the last province to regulate midwifery care in 2022 (PEI Midwives Association, 2024). In Canada, midwives are autonomous healthcare providers who provide antenatal, intrapartum, postpartum, and neonatal care. Midwives are a female‐dominated profession, often referred to as ‘experts in low‐risk birth’ (Neiterman, Lawford, and Borgeault 2017). Foundationally, the practice philosophies of midwifery emphasise provider‐patient collaboration, personalised care provision, informed consent, and choice of birthplace setting (CAM 2015).

Notably, many of the philosophical underpinnings of midwifery are in direct alignment with the Family Centred Maternity and Newborn Care principles outlined by the Public Health Agency of Canada (2017), CAM (2023). These guidelines aim to guide maternity care throughout Canada to ensure high‐quality, evidence‐based, culturally competent, and family centered care is available to all Canadians. Research shows midwifery reduces obstetric intervention rates, enhances birth experience satisfaction, and provides high‐quality maternal and newborn care to pregnant individuals with varying medical risks (Grybowski et al. 2015; Stoll et al. 2023; Mattison et al. 2020). Despite this, midwives are not adequately integrated throughout the Canadian healthcare system (Mattison et al. 2020; Thiessen, Haworth‐Brockman, Nickel, et al. 2020; Thiessen, Haworth‐Brockman, Nurmi, et al. 2020). Paradoxically, perhaps, this lack of integration seems particularly pronounced within rural and northern communities, which stand to benefit most from access to midwifery care. In Northern B.C., the percentage of births with midwives as the primary pregnancy provider is notably lower compared to other health authorities in the province (Stoll et al. 2023; Perinatal Services B.C. 2021). While B.C. leads in Canada's midwifery integration, accounting for 25% of all births being midwife‐led, only 80 out of 320 practicing midwives in B.C. are registered to work in rural areas, placing individuals in rural settings at a health access disadvantage with limited birth care provider choices (CAM 2023).

Living in northern and rural communities is recognised for its adverse effects on maternal and perinatal health services and outcomes (Canadian Institute for Health Information 2023a). However, defining ‘rural’ in a Canadian context poses challenges, as a clear global definition is lacking. Simply categorising Canada into rural and urban distinctions provides limited insight. Statistics Canada (2022a, 2022b) typically defines rural as areas outside Census Metropolitan areas (populations > 100,000) and Census Agglomerations (populations 10,000–99,999). Yet, this definition overlooks communities with fundamentally rural experiences, as some areas classified based on population size may have distinct health care needs due to geographic isolation and inequitable access. Considering accessibility to urban interfaces, many communities in northern Canada, including provincial northern regions, could be deemed rural despite population size, due to their limited access to urban centers. (Subedi, Roshanafshar, and Greenberg 2020).

In northern and rural communities, maternity service closures, lack of trained perinatal staff, and a decreasing number of family physicians providing maternity care services accentuate inequities for rural birthing individuals (Hedden et al. 2019; Kornelson 2024; MBAC 2014; Sutherns and Bourgeault 2008). Pregnant individuals living more than 2 h away from maternity services are more likely to be Indigenous and/or socioeconomically disadvantaged (Grybowski, Stoll, and Kornelsen 2011), compounding health inequities lived by rural women and their families. Indigenous scholars advocate for community‐based births to address the impact of colonisation, improve Indigenous maternal and neonatal outcomes, and reduce health inequities (Cidro, Bach, and Frohlick 2020; Duong 2019). Increasing the availability of midwives in northern and rural Canada is seen as crucial in promoting equitable access to maternity care (Stoll and Kornelsen 2014). However, to develop effective strategies to address the current lack of midwives in rural communities, it is necessary to examine what barriers exist for rural midwives when working in northern and rural areas.

## 2 The Review

This integrative review helps to address the rural midwifery gap by exploring what barriers midwives experience when practicing, or attempting to practice, in non‐urban communities. Presently, there is extensive literature exploring and addressing challenges in recruiting and retaining rural healthcare providers, such as physicians and nurses (Kulig et al. 2015; McDonald and Simpson 2013; Wilson et al. 2020), but there is a notable gap in literature specific to midwives. The challenges of sustaining rural maternity care are well documented in countries like the United Kingdom and New Zealand. This review adopts an international perspective, examining Canada alongside its Organisation for Economic Co‐operation and Development (OECD) peer countries to uncover shared barriers and highlight globally relevant insights into rural maternity sustainability (Canadian Institute for Health Information 2023b).

## 3 Aim

The purpose of this review is to better understand the specific barriers experienced by rural midwives to inform future research, practice, and policy initiatives directed toward increasing midwifery access in northern and rural communities.

## 4 Methodology

### 4.1 Design

We conducted an integrative review to examine the current barriers to rural midwifery practice. An integrative review is a broad approach that allows the researcher to combine both theoretical and empirical literature using a variety of data types and diverse research methodologies (Whittemore and Knafl 2005). The present review followed the five‐step process described by Whittemore and Knafl (2005), encompassing problem identification, literature search, critical appraisal as data evaluation, data analysis, and the presentation of results. The sources of data were empirical research papers, which, once analysed, were considered against Bronfenbrenner's Ecological Systems Theory (Bronfenbrenner 1994) at the micro‐, meso‐, and macro‐levels (Crawford 2020). Originally developed to study childhood development, the theory is widely used in health and social sciences to explain how multiple environmental systems influence individual experiences (Alam 2020; Noursi, Saluja, and Richey 2021). In Bronfenbrenner's framework, the microsystem refers to immediate social interactions, while the meso‐ and macrosystem describe the broader cultural and structural context, respectively.

### 4.2 Search Methods

After consultation with the University of Northern British Columbia (UNBC) health sciences librarian, in January 2024 a comprehensive literature search was undertaken in three databases: CINHAL Complete, Medline (Ovid), and Science Direct. Search terms and variations of search terms using Major Subject Headings (MeSH) and keyword searches included, but were not limited to: ‘midwifery’, ‘midwives’, ‘work experiences’, ‘practice’, ‘job experiences’, ‘barrier’, ‘challenge’, ‘obstacles’, ‘rural area’, ‘remote area’, ‘rural population’, ‘rural health’ and/or ‘rural health services’. These terms were searched with thr Boolean operator OR between variations of similar terms and searched with the Boolean operator AND between elements of the question. The search did not limit a date for articles, as challenges with rural maternity care in Canada have been well documented since the early 2000s (Grybowski et al. 2015). Table 1 outlines the inclusion and exclusion criteria used to identify the appropriate articles for review. All results from the searchers were first screened by title and abstract; a total of 58 articles received a full text review. Articles were excluded if they were not related to rural midwives' experiences, professional opinion, narrative or literature review, participants held dual registered nurse/registered midwife designation, the article did not differentiate between urban and rural experiences, there was specific health focus or intervention, or the article did not occur in an OECD peer country. A total of 14 articles met all inclusion criteria. See Figure one for the Prisma flow diagram regarding the selection of sources.

**TABLE 1:** Inclusion and exclusion criteria.

Inclusion criteria	Exclusion criteria
Countries including Canada, Australia, New Zealand, Sweden, the United Kingdom, Australia, Germany, France, the United States, and the Netherlands	Countries not Canada or a part of OECD peer countries
English language article	Sources focused on non‐urban pregnant individual experiences
Sources discussing barriers to rural midwifery practice	Sources focused on only urban midwifery experiences
Sources discussing rural midwifery practices or analysis of rural midwifery	Sources focused on specific health services Sources focused on other health care professionals in non‐urban settings

## 5 Findings

### 5.1 Search Outcome

Fourteen articles were identified as eligible for review, including seven qualitative (Siberry, Adams, and Leach 2023; Crowther 2016; Munro, Kornelsen, and Grybowski 2013; Olson and Couchie 2013; Brodie 2002; Gordon 1989), three quantitative (Martin and Reneau 2020; Hundley et al. 2007; Gordon and Erickson 1993), and four mixed methods (Dallenbach et al. 2020; Crowther et al. 2019; Gilkison et al. 2018; Kozhimannil, Henning‐Smith, and Hung 2016).

### 5.2 Quality Appraisal

Following Whittemore and Knafl's (2005) framework, critically appraising the articles was an important component, allowing for the evaluation of diverse sources. The Mixed Methods Appraisal Tool (MMAT) (Hong et al. 2018) was used to evaluate all the articles, as it is an ideal tool designed for the appraisal stage of reviews of multi‐methodologies. It allows for evaluation criteria specific to each study type, enabling a standardised and robust assessment of the overall quality of the research (Hong et al. 2018). Using the MMAT tool, the included studies were all found to be of medium to high quality. No records were excluded following critical appraisal.

### 5.3 Data Abstraction and Synthesis

Data was independently extracted using an integrative literature review (ILR) matrix; see Table 2. Data was abstracted regarding the country of origin, the research question, methodology, sample number, and type of participants. Then the studies were examined to summarise their findings, highlighting barriers to rural midwifery practice and rural midwifery experience.

**TABLE 2:** Integrative literature review matrix (IRL).

Author and date	Research question	Country	Methodology	Sample number (*n*) & type of participants	Summary
Gordon (1989)	Assess the effects that malpractice insurance has on CNMs practicing in Arizona's rural countries	United States of America	Exploratory descriptive Qualitative Methodology	Certified nurse midwives (*n* = 21)	Although midwifery is rising in demand in Arizona, the practice autonomy of midwives has decreased. Despite a rising attendance of midwives at births, the number of rural midwives did not increase. Malpractice insurance needs by physicians and midwives negatively affected the integration and sustainment of rural midwifery
Gordon and Erickson (1993)	A survey of certified nurse‐midwives in Arizona was undertaken to provide data for maternity services planning	United States of America	Quantitative Descriptive	Certified Nurse Midwives Urban (*n* = 32) Rural (*n* = 10) Not practicing (*n* = 18)	Lack of access to health services is a major concern in rural America. Certified Nurse (CNM) midwives may be able to help ensure adequate health service access for families in childbearing years. Recruiting and retaining CNM's is complex, and their study found that CNM's who lived in rural areas were more likely to want to work in those areas. CNM's working in rural communities found that physicians and hospital admitting privileges were barriers to practicing in rural areas. They recommend that rural communities recruit students from rural areas and offer midwifery education programs locally
Brodie (2002)	What are the barriers to the provision of safe and efficient maternity services in Australia? What are the strategies to overcome these barriers?	Australia	Exploratory descriptive Qualitative Methodology	(*n* = 563) 73% current practicing midwives. 28% rural midwives	Midwifery is under‐resourced, depleted in numbers, and is an under‐recognised profession. Significant concerns were illuminated that led to midwives being unable to fulfil their full role in maternity services within Australia. While this article is not limited to rural midwifery, it highlights several key features that are unique and significant for rural midwives
Hundley et al. (2007)	Is the competence and confidence of midwives affected based on practice location (rural vs. urban)	Scotland	Quantitative, analytical cross‐sectional design	Total (*n* = 189) Rural Midwives (*n* = 82) Urban Midwives (*n* = 107)	While it was previously assumed that rural midwives were less confident and competent in their practice, the findings from this study suggest that the issue of competence is far more complex and deserves further attention
Munro, Kornelsen, and Grybowski (2013)	What are the barriers and facilitators to interprofessional models of maternity care between physicians, nurses, and midwives in rural British Columbia	Canada	Qualitative, exploratory framework	Total (*n* = 73) Midwives (*n* = 7) Physicians (*n* = 27) Nurses (*n* = 18) Birthing Women (*n* = 5) Community‐Based Providers (*n* = 5) Administrators (*n* = 5) Decision Makers (*n* = 5)	Interprofessional collaboration is often seen to improve midwifery access in rural and remote communities; however, there are tensions within interprofessional collaboration. These include negative perceptions of midwifery and homebirth, pay structure differences, and scope differences
Olson and Couchie (2013)	To explore the role of midwives in the implementation of an elective birthing program in one remote First Nation community in Canada To identify current barriers to the challenges of the practice of midwifery in these settings	Canada	Multisided ethnography based on 15 months of fieldwork in Manitoba, Canada	Total (*n* = 39) Interviews included pregnant Aboriginal women, fathers, grandmothers, First Nations political leadership, policy makers, hospital nurses, community nurses, First Nations women, doctors, and midwives	Place of birth is central to the topic of Indigenous maternal healthcare in Canada. Midwifery should be central when moving birthing services back to rural and remote Indigenous communities. The implementation of rural midwifery can address the goal of having a skilled birth attendant at every birth and filling the gap that exists within the current system that forces evacuation from home community for birth
Kozhimannil, Henning‐Smith, and Hung (2016)	Describe the role of certified nurse‐midwives in providing maternity care in rural U.S. hospitals	United States of America	Mixed Methods, Concurrent design Qualitative Descriptive Approach And Quantitative descriptive analysis	Hospitals (*n* = 263)	This study highlighted the growing midwifery profession within rural communities in the United States. They found that enabling autonomous practice of midwives increased the number of midwives available for practice in rural communities
Crowther (2016)	Examines how relationships influence safety in rural maternity care	New Zealand	Interpretive Hermeneutic Phenomenology	Total (*n* = 13) Mothers (*n* = 3) Midwives (*n* = 6) Ambulance crew (paid & unpaid) (*n* = 3) General Practice OB (*n* = 1)	Relationships are vital within maternity care, and relationships are founded on mutual understanding. Trust builds healthy rural communities of practice where all healthcare professionals can flourish and create safe birthing opportunities for rural families. Discord between midwives and other healthcare providers can be harmful and make practice unsustainable
Crowther (2016)	Discover the financial lived experiences of rural midwife participants	New Zealand	Qualitative hermeneutic phenomenology design	Rural and remote midwives (*n* = 6)	Rural midwives are running small businesses' that can be seen dually as an ‘expensive hobby’. Many midwives are simply surviving in small rural communities, and that funding is uneven for some rural and remote midwives
Gilkison et al. (2018)	Explore and identify the range of skills, qualities, and professional expertise needed for rural midwifery practice in New Zealand and Scotland	New Zealand and Scotland	Mixed Methods Concurrent design Qualitative Descriptive Approach and Quantitative descriptive analysis	Quantitative: (*n* = 222) Scotland Midwives (*n* = 77) New Zealand Midwives (*n* = 145) Qualitative: (*n* = 15) Scotland (*n* = 3) New Zealand (*n* = 12)	Rural midwives in New Zealand and Scotland have courage and fortitude as a unique skill that is required to underpin practice. Midwives who choose to work in rural communities enhance skills such as preparedness, resourcefulness, and developing meaningful relationships to safeguard birth
Crowther et al. (2019)	Explore the practical realities of midwives working in remote and rural areas	New Zealand and Scotland	Mixed methods Concurrent design. Qualitative descriptive approach and Quantitative descriptive analysis	Quantitative: (*n* = 222) Scotland Midwives (*n* = 77) New Zealand Midwives (*n* = 145) Qualitative: (*n* = 15) Scotland (*n* = 3) New Zealand (*n* = 12)	Rural midwifery practices require many differing levels of collaboration with other healthcare providers. These relationships can be built and sustained over time; however, they are often not easy to establish and require effective communication skills and social building capital
Dallenbach et al. (2020)	Gain knowledge to inform optimisation of equitable and sustainable maternity care for rural communities	New Zealand	Mixed methods: Exploratory sequential design quantitative descriptive analysis and followed by qualitative descriptive approach	Quantitative (*n* = 145) Qualitative (States: a subset; however, *n* not given)	Rural midwives face economic, geographical, meteorological, and workforce challenges. This increases the sense of vulnerability within the profession. However, rural midwives can draw strength to provide high‐quality maternity care for rural women. Interprofessional relationships are crucial in rural maternity care
Martin and Reneau (2020)	To focus on the structure and individual components of collaborative practice agreements, which define the scope of practice of an NP or CNM and to what degree they are permitted to practice under general or direct supervision by a clinician	United States of America	Quantitative descriptive	Total (*n* = 1178) Nurse Practitioners (*n* = 675) Certified Nurse Midwives (*n* = 503)	Due to the impending workforce shortage that impacts communities' access to quality perinatal, neonatal, and primary care services, advanced practice registered nurses (APRN), including nurse practitoners and certified nurse midwives, need to be able to practice to the full extent of their education and training. The study reported on the existing barriers to APRN's ability to practice in women's health settings, helping to inform changes that need to occur at the legislative level. APRNs can make vital health care services more accessible by working sideby side with physicians instead of under them
Siberry, Adams, and Leach (2023)	How do Ontario midwives' interprofessional relations vary across the urban and rural hospital contexts?	Canada	Grounded theory Qualitative descriptive	Midwives (*n* = 26) Rural (*n* = 10) Urban (*n* = 16)	Interprofessional collaboration is essential to teamwork in both urban and rural settings. Rural midwives have unique working experiences where they generally have collegial relationships with healthcare professionals. However, rural physicians still lacked an understanding of the rural midwife role, which hindered the integration of rural midwives. Urban midwives experienced higher levels of conflict related to ‘turf war’ in the clinical setting. In both settings, conflicts occurred regarding imposed limits to midwives' scope of practice and physician oversight in hospital privileging. Hospital leadership is essential to mitigating these tensions

Aligning with the approach described by Torraco (2016), the reshaping of the data by tabulating, comparing, combining, and looking for patterns can help with data analysis and synthesis. In addition, a thematic analysis approach was used to identify overarching themes emerging from the results of the included articles (Braun and Clark 2021). Using an inductive, iterative approach, initial coding generated themes that are more descriptive in nature. As coding progressed, higher‐level themes emerged, with some themes collapsing into each other while others became more refined. In the final stages of analysis, themes were explored and grouped using the Ecological Systems Framework with the goal of identifying barriers across the micro‐, meso‐, and macro‐levels of rural midwifery practice.

## 6 Results

### 6.1 Summary of Included Articles

The 14 articles included in the review ranged from 1990 (oldest article) to 2023 (newest article). Eight of the included articles were within 10 years of the search date, and two were within 11 years, published in 2013. The final four included articles spanned between 1990 and 2007. The included articles originated across several OECD countries. Four articles originated from the United States of America, three from Canada, three from New Zealand, one from Scotland, and one from Australia. Lastly, two articles explored rural midwifery experiences in both New Zealand and Scotland.

### 6.2 Thematic Analysis

Aligned with the Ecological Systems theory, the findings of the thematic analysis are arranged by viewing barriers as experienced by midwives at the micro‐level, meso‐level, and macro‐level. Three higher‐level themes were identified; these included: (1) Micro Barriers: The Challenge of Living the Life as a Rural Midwife, with subthemes of *professional isolation, financial uncertainty*, and *being known*; (2) Meso Barriers: Career Stressors and Strains, with subthemes of *continuing education, interprofessional relationships*, and *the urban/rural interface;and* (3) Macro Barriers: The System is Not Set Up for Rural Midwifery, with subthemes of *professional recognition, the urban biomedical model of care*, and *policy impacts practice*.

### 6.3 Micro Barriers: The Challenge of Living Life as a Rural Midwife

#### 6.3.1 Professional Isolation

The nature of birth work is notably unpredictable. Seven of the articles included in this review explored how the work conditions of on‐call birth work were intensified for rural and remote midwives. Gilkison et al. (2018) noted that to be successful, rural midwives needed mental and emotional stamina. The intensity of birthing work takes endurance, which is problematic when midwives work independently, potentially as a single provider in a community. For many midwives, there was often no access to adequate backup, which created a sense of isolation and loneliness (Crowther et al. 2019; Gilkison et al. 2018; Gordon 1989). The sense of isolation was intensified both physically and emotionally, especially when the rural midwife needed assistance and had unstable or inadequate relationships with other local healthcare providers, such as registered nurses (RNs) or family physicians (Crowther 2016). Recognising that isolation can take an emotional toll, some midwives have explored team‐based or shared care approaches to prenatal care (Munro, Kornelsen, and Grybowski 2013). While such strategies have been utilised by rural midwives to improve working conditions, there are challenges. Shared care often means that rural midwives needed to take on increased postpartum care work while remaining on call for home births during the physician's call week, which created immense challenges for midwives to maintain an adequate work‐life balance (Munro, Kornelsen, and Grybowski 2013).

#### 6.3.2 Financial Uncertainty

Low volume of deliveries in rural areas coupled with case load variability often meant that rural midwives faced financial uncertainty (Dallenbach et al. 2020; Crowther 2016; Gordon 1989; Gordon and Erickson 1993). There was limited consideration of financial remuneration for rural midwives in relation to transit times. Rural midwives often needed to travel long distances to appointments and births or travel with patients when transferring to higher levels of care (Crowther 2016; Dallenbach et al. 2020). These vast distances in rural communities lead to higher vehicle mileage and maintenance, where the financial onus was on the midwife (Crowther et al. 2019). Weather conditions and availability of rural paramedic services often complicated transfers and led to the increased risk of being stranded with no way to return home, often relying on the kindness of strangers or relying on their families to come to retrieve them (Crowther et al. 2019; Crowther 2016; Dallenbach et al. 2020).

#### 6.3.3 Being Known

Midwives in small communities, ‘where everybody knows everybody’, have a difficult time separating their personal identity from their professional one (Crowther et al. 2019; Dallenbach et al. 2020; Gilkison et al. 2018). Rural midwives care for women from diverse backgrounds and complicated social situations, requiring them to be adaptable, flexible, and non‐judgemental (Gilkison et al. 2018). They must often work beyond the traditional role of the midwife due to the lack of other services in rural communities, such as lactation consultants, counselling, or taxi services (Crowther 2016; Gilkison et al. 2018). Working as the consistent or only point of perinatal contact and living in tight‐knit communities increased the intensity of the midwife‐patient relationship. Crowther (2016) noted that in the context of being known, they are personally linked to both good outcomes and bad outcomes of birth within the community. These factors prohibited personal privacy and the ability for the midwife to ‘turn off’ their midwifery role at home, which further exacerbates challenges in maintaining an adequate work‐life balance (Crowther et al. 2019; Gilkinson et al.).

### 6.4 Meso Barriers: Career Stressors and Strains

#### 6.4.1 Continuing Education

The ability to maintain skills as a rural practitioner was identified in several articles as a barrier to continuing midwifery practice. Challenges related to the availability and location of the continuing professional development were underscored by issues of geographical isolation (Hundley et al. 2007; Crowther 2016; Brodie 2002). Finding coverage to attend continuing education, as well as the financial burden of paying for continuing education, was a barrier for rural midwives (Crowther 2016; Crowther et al. 2019; Hundley et al. 2007). Further, rural midwives had to be able to meet their full scope of practice and extend their training to the complex demands of low‐resource rural care. Rural midwives required unique skills and abilities to anticipate and recognise problems when being hours away from secondary services (Gilkison et al. 2018). This may help to explain the contrasting findings by Hundley et al. (2007), who found that despite the barriers to attending continuing education, self‐reported confidence was higher among rural midwives.

#### 6.4.2 Interprofessional Relationships

Many of the articles in the review highlighted that midwives experience interprofessional strain in their relationships with other healthcare professionals, such as physicians and nurses (Brodie 2002; Crowther et al. 2019; Crowther 2016; Dallenbach et al. 2020; Gordon and Erickson 1993; Munro, Kornelsen, and Grybowski 2013; Olson and Couchie 2013). In settings where midwifery is unfamiliar, there was resistance to changing the current health services model to accommodate midwifery due to stigmatisation over the midwifery birth philosophy (Brodie 2002; Olson and Couchie 2013).

Physician resistance to midwifery was a common theme within several articles (Gordon and Erickson 1993; Gordon 1989; Munro, Kornelsen, and Grybowski 2013; Crowther 2016; Siberry, Adams, and Leach 2023). Rural physicians feared that adding midwifery would impact their sustainability, finances, or insurance rates, reporting they would be inclined to leave rural communities without adequate maternity volume (Gordon and Erickson 1993; Munro, Kornelsen, and Grybowski 2013). In addition, labour and delivery nurses expressed frustration around the conflict of roles and responsibilities and noted that communication issues created tensions between midwives and nurses (Munro, Kornelsen, and Grybowski 2013). Alternatively, one study found that rural midwives experienced less interprofessional tension and found it easier to integrate into smaller hospitals compared to larger urban settings. (Siberry, Adams, and Leach 2023). However, within this study, tensions between rural midwives and rural family physicians were still prominent. The discord was underpinned by a lack of understanding of the safety of midwifery and ideological differences when approaching birth (Crowther 2016; Munro, Kornelsen, and Grybowski 2013; Olson and Couchie 2013).

Midwives also reported they felt they had to ensure they built adequate relationships with community agencies, such as police, social services, and paramedic services (Crowther et al. 2019). Similar to the micro‐level theme of being known, rural midwives experience unique challenges when balancing their personal and professional identity in small, close communities. Midwives must navigate the delicate balance between maintaining patient confidentiality and trust while fostering positive relationships with police and social services in rural communities where there may be social or personal overlap. This long‐term trust is critical for addressing sensitive issues, such as domestic violence or child protection concerns, to ensure the safety and well‐being of mothers and newborns (Crowther et al. 2019).

Due to the unpredictable nature of childbirth, paramedic services play a crucial role in facilitating transfers from home to the hospital or from rural to urban settings in dynamic situations. However, rural midwives reported experiencing professional exclusion in quality improvement discussions regarding maternal transfers between general practitioners and ambulance services or had to fight for the right to receive support from medical transport teams (Crowther 2016; Olson and Couchie 2013).

#### 6.4.3 The Rural/Urban Interface

Urban counterparts lacked understanding and appreciation for rural practice (Crowther et al. 2019; Crowther 2016; Dallenbach et al. 2020). Midwives reported feeling unsupported when they arrived at larger centres, often facing criticism from urban colleagues regarding the care they provided or the decisions they made. These criticisms failed to account for the challenges inherent in rural settings, where resources are rationed and support services are scarce (Crowther et al. 2019; Dallenbach et al. 2020). At times, midwives reported issues began as soon as they initiated the call for transport (Dallenbach et al. 2020). Rural midwives reported experiences of spending time in dialogue with urban counterparts in order to justify the reasons for delayed transfer, which impacted the urgency of the current situation (Crowther et al. 2019; Crowther 2016; Dallenbach et al. 2020). Crowther (2016) noted that decision‐making and communication between the urban/rural interface needed to be courteous, tactful, and focused on the well‐being of the patients, rather than judgemental or accusatory.

### 6.5 Macro Barriers: The System Is Not Set Up for Rural Midwifery

#### 6.5.1 Professional Recognition

There was a noted lack of formal professional recognition for midwives, which compounded the discord in interprofessional relationships that was noted at the meso level (Munro, Kornelsen, and Grybowski 2013; Dallenbach et al. 2020; Brodie 2002). There was an overt medical dominance placing an emphasis on physician models of maternity care and nursing (Brodie 2002; Crowther 2016), and midwifery representation was lacking in leadership roles in communities (Brodie 2002). The impact of a lack of professional recognition was noted within several articles, such as physician‐led advisory boards maintaining control of granting midwifery hospital privileges and determining their scope of practice (Munro, Kornelsen, and Grybowski 2013; Siberry, Adams, and Leach 2023). Brodie (2002) related this resistance to midwifery to the elevated level of medical dominance within health care systems.

#### 6.5.2 Urban, Biomedical Model of Care

Rural midwifery success was perceived to be more likely in areas with higher volumes of deliveries (Kozhimannil, Henning‐Smith, and Hung 2016). Financial remuneration for midwives was reported to be based on an urban model, where practice groups were well‐staffed and geographical areas were more compact (Dallenbach et al. 2020; Crowther 2016; Brodie 2002). Conventional, urban based recruitment strategies had little impact on engaging midwives to work in rural, underserved areas (Gordon and Erickson 1993; Kreiner 2009), and individuals from rural areas were more likely to return to work in rural communities (Gordon and Erickson 1993). Finally, it was explored that urban centres created the standards of care; many rural centres could not achieve this, and it exacerbated the challenges in maintaining maternity services in rural areas (Brodie 2002).

#### 6.5.3 Policy Impacts Practice

Government policies significantly impact the success of midwifery, particularly in rural areas (Kozhimannil, Henning‐Smith, and Hung 2016; Brodie 2002; Kreiner 2009). Requirements for malpractice insurance and collaborative practice agreements create financial and operational barriers for midwives, complicating their ability to establish and maintain practices (Gordon 1989; Gordon and Erickson 1993; Martin and Reneau 2020). Midwives viewed these policies as worsening the strained relationships between midwives and physicians, hindering collaborative care. In Olson and Couchie's (2013) study, they explored how a lack of clear governmental responsibility, especially between federal and provincial levels, similarly obstructed establishing midwifery practice. Strong policy support at all levels was seen by midwives as essential for enabling them to deliver high‐quality maternal and newborn care (Brodie 2002; Martin and Reneau 2020; Munro, Kornelsen, and Grybowski 2013; Olson and Couchie 2013).

## 7 Discussion

The results of this review illuminate that there are considerable barriers present at the micro‐, meso‐, and macro‐levels affecting the integration and sustainability of midwifery services in rural communities. It is notable that many of the micro‐ and meso‐level barriers appear to be a consequence of macro‐level, systematic barriers, yet tensions at the micro‐ and meso‐level sustain and reinforce the power imbalance at the macro‐level. At the micro‐level, the long, unpredictable hours, coupled with a lack of backup and support and financial variability, create a state of ‘just surviving’ in rural practice. This may make it difficult for rural midwives to organise and advocate for themselves and systems changes (Crowther 2016; Crowther et al. 2019). This leaves urban organisations and structures to prioritise the needs of rural communities and midwives, without a full understanding of the unique realities and constraints of rural practice. At the meso level the biomedical dominance of healthcare compounds the tensions in relationships between midwives and other healthcare professionals (Sattar et al. 2023; Wranik and Haydt 2018). The prevalence of biomedical practices intensifies the conflicts in the interactions between midwives and other health care practitioners. These conflicts contribute to the ongoing power struggle between the biomedical model and midwifery ideologies, ultimately resulting in an imbalance of power, where those striving to gain or maintain authority wield unequal influence.

Within the review, there was a noted lack of representation for midwives, with few midwives in leadership roles, thereby affecting the visibility of midwifery in rural planning services. Despite midwifery's reintroduction in Canada and efforts to enhance accessibility, family physicians remain the primary providers of prenatal care and attendants at births (Benoit et al. 2010). A concerning decline in primary care physician attendance, especially in rural areas, poses risks and limits choices for families (Hedden et al. 2019). Notably, there is no visible midwifery representation within Canada's Ministry of Health. Addressing leadership pluralism is essential, especially given the recent 2022 appointment of a Chief Nursing Officer in the Ministry of Health, highlighting the importance of representation from all relevant professions in health system planning and advocacy.

The under‐representation of midwives in key leadership roles contributes to the absence of clear and supportive policies, affecting midwifery integration, practice, and sustainability. The slow, formal reintroduction of midwifery in Canada exemplifies how policy influences midwifery integration and sustainability. The Yukon and PEI were the last territory and province to regulate midwifery in 2021 and 2022, respectively. Another notable example included within the review is from Olson and Couchie (2013) study and is particularly relevant for the Canadian context. Their study demonstrates the essential impact of a lack of clear policy on the maintenance of midwifery in rural and remote communities in Canada. Government agencies met the community in question with red tape processes and a refusal to assume responsibility for ensuring safe birth within the community. In this case, an Indigenous community lost access to local birthing services, perpetuating the cycle of forced evacuation for childbirth. However, such outcomes are not inevitable for rural communities. With adequate support, rural midwifery in Australia, Canada, and New Zealand has proven effective in expanding birthing options, enabling families to remain closer to home, increasing prenatal care utilisation, and reducing rates of mortality and morbidity (Bacciaglia et al. 2023; Gibbons et al. 2016; Kolahdooz et al. 2016; Van Wagner et al. 2012).

Rural and northern families face significant healthcare access challenges, exacerbated by the dominance of the biomedical model and urban‐focused care systems. The biomedical model is dominant in Canada and other OECD countries. In the biomedical model, physicians assert the most influence on the administration of health care (Wranik and Haydt 2018). Historically this influence has led to restricting the autonomy and integration of other healthcare professionals—like midwives (Zaman 2017). As a result, rural service planning often focuses on urban, physician‐led strategies that fail to reflect the realities and needs of rural communities (Behruzi et al. 2017; Benoit et al. 2010; Wilson et al. 2020).

Urban‐focused, biomedical dominance contributes to a cycle of disempowerment for midwives, who are a predominantly gendered profession. This can limit career advancement opportunities and reinforce power imbalances (Pincha Baduge et al. 2024). This aligns with broader global concerns regarding systemic barriers faced by heavily gendered professions, including midwifery and nursing (Benoit et al. 2010; WHO 2021). While not directly examined within the review, this is echoed in the findings where it was noted that midwifery representation was lacking in leadership and health planning roles.

Within this integrative review, there are several limitations to acknowledge. The search for articles did not include a limit to the age of articles, given the long‐standing challenges to rural midwifery integration. Allowing an unrestricted date range for the literature search can result in the inclusion of outdated information, which might misalign the study's conclusions with current practices. However, it is notable that many of the themes that arose within the older articles were supported by information within the newer, more recent literature. This warrants an examination of why these challenges for midwives have persisted.

Another limitation of this review is the lack of sources that originated within Canada, with the majority of articles originating from OECD peer countries, including New Zealand, Australia, the United States of America, and the United Kingdom. However, while this may limit the direct applicability of the findings to the Canadian context, the barriers described by rural midwives in New Zealand, Australia, the United States, and the United Kingdom have clear practical relevance across diverse settings and thus provide valuable insights that can inform understanding of similar challenges in the Canadian context.

The primary author generated and reviewed the themes, albeit discussed at length with supervisors. This creates the potential that the individual perspective or preconceived notions may influence the interpretations drawn from this data. While conducting this integrative review, I acknowledged my background as a registered nurse, woman, mother, and settler, and that my prior experiences of working in maternal child health have shaped my lens in how I approached and analysed the data. However, I remained conscious of the need for objectivity and strived to mitigate personal biases by employing rigorous methodologies, seeking diverse perspectives in the research, and critically evaluating the findings.

## 8 Conclusion

There are many barriers to the sustainability of rural midwifery practice that can be identified at the micro‐, meso‐, and macro‐levels. The findings from this review suggest that to improve rural communities' access to midwifery services, rural planning committees and policymakers need to explore how to increase midwifery representation, improve policies and laws related to midwifery care services, and move away from the overt medical, urban model of healthcare when planning for rural maternity sustainability. A paucity of Canadian literature suggests a need for research into how and why midwifery struggles to be integrated into the rural setting and which strategies need to be explored to overcome barriers. Midwifery has demonstrated success in improving access to culturally safe care, enhancing prenatal care utilisation, and reducing mortality and morbidity for underserved populations, such as those living far from maternity services (Bacciaglia et al. 2023; Kolahdooz et al. 2016; Van Wagner et al. 2012; Grybowski, Stoll, and Kornelsen 2011). To address these inequities, healthcare systems must integrate midwifery as an equal profession, fostering collaboration between midwives and physicians (Martin and Reneau 2020).

## Conflicts of Interest

The authors declare no conflicts of interest.

## Peer Review

The peer review history for this article is available at https://www.webofscience.com/api/gateway/wos/peer‐review/10.1111/jan.16737.

## Data Availability

Data sharing not applicable to this article as no datasets were generated or analyzed during the current study.
